# Hydropneumothorax as a Presentation of Birt-Hogg-Dubé Syndrome

**DOI:** 10.7759/cureus.38465

**Published:** 2023-05-02

**Authors:** Anand Dhaliwal, Nancy Le, Daniel I Razick, Muzammil Akhtar, Biljinder S Chima

**Affiliations:** 1 Surgery, California Northstate University College of Medicine, Elk Grove, USA; 2 Neurology, California Northstate University College of Medicine, Elk Grove, USA; 3 Family Medicine, Sports Medicine, Rocklin Family Practice and Sports Medicine, Rocklin, USA

**Keywords:** renal cancer, fibrofolliculomas, hydropneumothorax, birt-hogg-dubé syndrome (bhds), autosomal dominant genetic disorder

## Abstract

Birt-Hogg-Dubé syndrome (BHDS) is a rare genetic disorder characterized by cutaneous, pulmonary, and renal manifestations. We present a unique case in which a combination of multiple chronic illnesses, genetic testing, and significant family history led to a diagnosis of BHDS. A 72-year-old male patient presented to their primary care physician with a persistent cough of four months and was admitted to the emergency department after the discovery of a left hydropneumothorax. The patient's medical history was significant for recurrent spontaneous bilateral pneumothoraces diagnosed over 20 years ago, chronic obstructive pulmonary disease (COPD), and many other systemic illnesses. A combination of the patient's significant past medical and family history led to a diagnosis of BHDS. Genetic testing was also done to confirm the diagnosis. Despite benign skin lesions being the most common finding, they are not always present, as seen in our case, which can result in missed diagnosis. Due to the autosomal dominant nature of BHDS, it is vital to make an accurate diagnosis to allow for proper genetic counseling, as the development of renal cancer is the leading cause of mortality.

## Introduction

Birt-Hogg-Dubé syndrome (BHDS) is a rare, autosomal dominant condition, which arises from mutations in the folliculin (*FLCN*) gene [1]. *FLCN* is a tumor suppressor gene on chromosome 17, which encodes the folliculin protein [2]. While presentation is variable, BHDS is commonly characterized by the formation of fibrofolliculomas, spontaneous pneumothorax, pulmonary cysts, and an increased risk of developing renal cancer [3]. Despite the major genetic implications, BHDS can present with any combination of pulmonary, skin, or renal manifestations even within the same family [4]. While extremely rare, de novo development of the disease has been recorded [5]. The prevalence of BHDS has been estimated to be two cases per million people with no gender differences [4]. 

Given the multifaceted nature of phenotypic presentation for BHDS, diagnosis can be complicated. While skin lesions are typically the first clinical manifestation, pulmonary disorders can also present initially as seen in our patient. However, if left undiagnosed, renal cancer can be a long-term, yet devastating, consequence. It is estimated that BHDS patients are at a sevenfold increased risk for the development of bilateral and multifocal renal tumors [6]. As with most neoplastic disorders, renal cancer survival depends greatly on the stage at diagnosis. However, the metastatic form of the disease has a 12% five-year survival rate, making early diagnosis crucial [7]. Therefore, it is imperative that providers take a thorough history and perform genetic testing promptly if the condition is suspected, and routinely screen for renal cancer if the mutated FLCN gene is found.

We present a unique case of BHDS in which diagnosis was made based on a combination of a left hydropneumothorax, a history of bilateral pneumothorax, absence of fibrofolliculoma skin lesions, and genetic testing showing a mutation in the *FLCN *gene. 

## Case presentation

A 72-year-old male patient presented to his family medicine physician for an annual wellness visit, during which analysis of the patient’s past medical and family history led to suspicion of BHDS. Despite presenting with actinic keratosis and posterior right shoulder pain, the patient’s vitals and physical exam findings were otherwise normal.

His past history included recurring bilateral pneumothoraces with apical blebs 20 years ago, requiring bilateral placement of chest tubes. Pleurodesis was successful in the right lung, but unsuccessful in the left as the patient could not tolerate the pain. Additionally, 14 years prior, the patient was diagnosed with chronic obstructive pulmonary disease (COPD), calculus of the kidney, cardiac murmurs, benign prostatic hyperplasia without urinary obstruction, and essential hypertension. The patient also had a history of smoking two packs of cigarettes a day for 10 years, which he quit at the age of 27.

**Figure 1:**
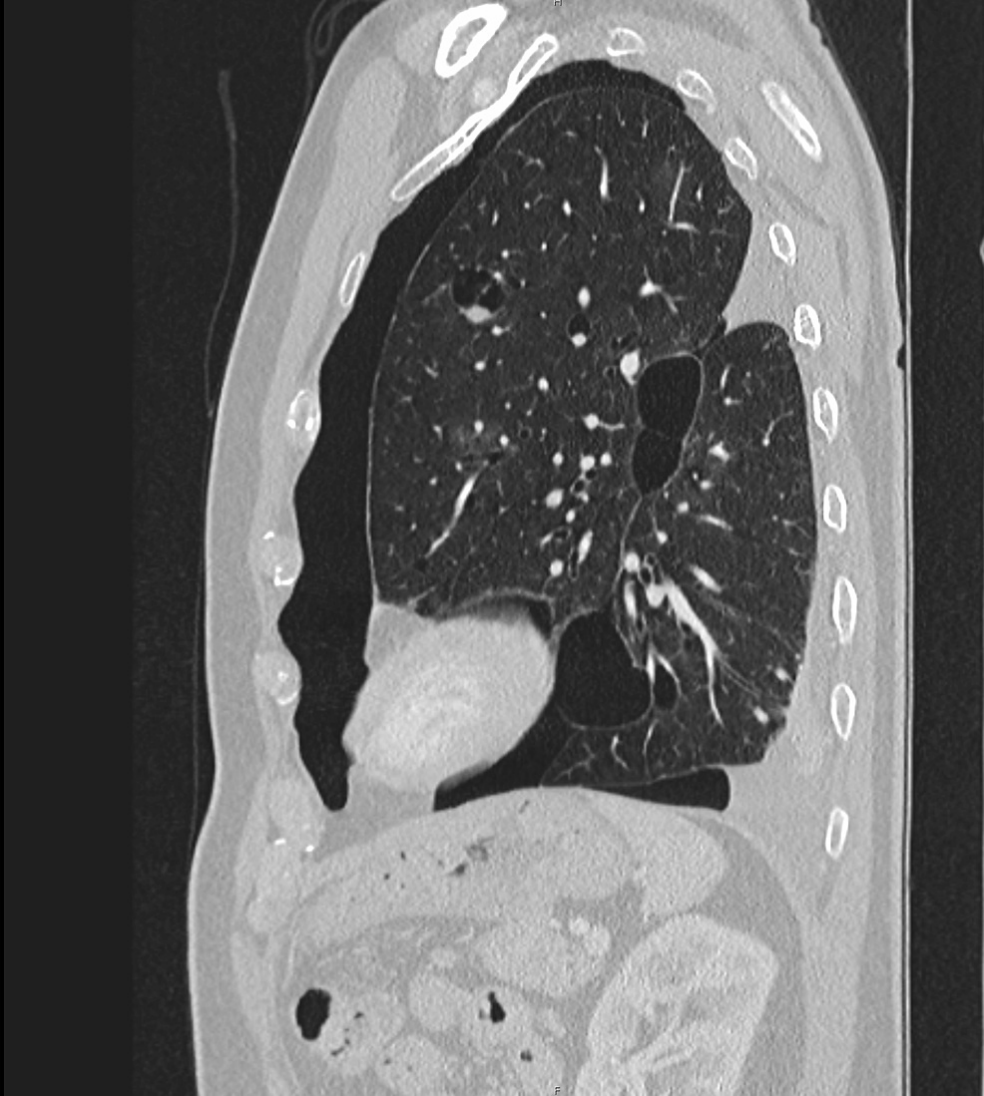
Computed tomography (CT) scan of left lung revealing hydropneumothorax; this was from nine months prior to the current visit when the patient presented with four months of persistent cough, shortness of breath, and a cold

Nine months ago, the patient presented to his primary care physician with a persistent cough, shortness of breath, and cold for the past four months. Computed tomography (CT) of the chest led to the discovery of a left hydropneumothorax. The hydropneumothorax extended from the apex along the left lateral chest wall down to the diaphragm, and there was the presence of air-fluid level along the diaphragm (Figure 1). The left upper lung was hyperlucent with basilar platelike opacity. The patient was subsequently admitted to the hospital for four days as he required placement of a chest tube. Despite denying pleurodesis of the left lung, the patient’s symptoms improved; however, he still had a mild cough which was triggered by talking when he presented to his primary care physician a week later for follow-up.

A month prior to his admission to the hospital, the patient was placed on one puff of fluticasone/umeclidinium/vilanterol (Trelegy Ellipta) every morning for maintenance treatment of his COPD symptoms. 

The patient’s family history was significant for his mother being diagnosed with lung cancer, his father with emphysema and alcoholism, and his paternal uncle with colon cancer. During his most recent wellness visit, a full sequence analysis of the *FLCN* gene’s entire coding region was ordered based on suspicion of BHDS, given the patient’s significant history of recurrent pneumothorax. Results revealed a pathogenic variant in one copy of the *FLCN*, allowing for a final diagnosis of BHDS to be made (Table 1). 

**Table 1:** Genetic test revealing pathogenic variant in folliculin gene Note: A pathogenic variant was identified in one copy of the *FLCN* gene, which is consistent with a diagnosis of Birt-Hogg-Dube (BHD) syndrome.

FLCN			
Variant 1	See note		
c.1252del (p. Leu418Trpfs*50)			
Classification 1	See note		
Pathogenic			
Lifetime risk 1	See note		
Path variant			
Interpretation	Positive		

## Discussion

BHDS is a complex autosomal dominant condition classically characterized by various combinations of skin fibrofolliculomas, pulmonary cysts, spontaneous pneumothorax, and renal cancer. Germline mutations in the *FLCN* gene, which encodes for the protein folliculin, have been identified as the underlying genetic cause of the disease. While the exact function of the protein is still not fully understood, it has been linked with the mammalian target of the rapamycin (mTOR) pathway [8]. Deregulation within the mTOR pathway is a commonly identified alteration in human cancers, as it is involved in cell growth and proliferation, thereby suggesting its role in the pathogenesis of BHDS [9]. Dermatologic involvement in the form of fibrofolliculomas is typically the first and most frequent manifestation of BHDS, appearing in the third decade of life in nearly 90% of patients. These lesions present as round, white-gray papules 1-4 mm in size and they most often affect the midface, though they can develop anywhere on the head and neck [10]. Bilateral pulmonary cysts are present in approximately 70-80% of patients, of which 30% develop single or recurrent episodes of spontaneous pneumothorax, typically before the age of 40 [11]. Renal cancer is commonly a later clinical manifestation found in 12-34% of patients around the age of 50, with the timing of diagnosis ranging from 30 years to 70 years. BHDS is associated with various renal tumors including chromophobe tumors, hybrid chromophobe/oncocytic tumors, and clear cell carcinoma [12]. Compared to the classical findings in BHDS, the patient, in this case, had several unique findings including the first reported hydropneumothorax associated with the disease, absent fibrofolliculomas, recurrent bilateral pneumothorax, and late-onset actinic keratosis. Though actinic keratosis may simply have been a coincidental finding, it is possible that BHDS may have been the underlying cause. We present these unique findings of BHDS so clinicians may be aware of atypical presentations that may manifest in this rare disease, allowing for timely diagnosis and establishment of a care plan.

The most severe manifestation of BHDS is typically considered to be some form of renal cancer, which usually presents later in the course of BHDS. This highlights the importance of early diagnosis and appropriate management to prevent fatal metastases. Benusiglio et al. found that in a group of 33 BHDS patients who presented with renal tumors with 21 varying mutations in the *FLCN* gene, the age at which the tumor presented was highly variable [13]. Therefore, the onset of renal involvement cannot simply be considered as a late manifestation of BHDS, highlighting the importance of its suspicion in even young patients as early as in their 20s or 30s. When patients are diagnosed with or suspected to have BHDS, it is vital to perform frequent abdominal and pelvic examinations to detect possible renal involvement, in addition to an assessment of other BHDS symptoms. While our patient did not have renal cancer, he had a 14-year history of kidney stones. Given the fact that Benusiglio et al. found 21 different mutations in the *FLCN* gene in only 33 patients, variation in mutations could potentially be a cause of atypical presentations as seen in our patient [13].

In the first known incidence of BHDS in 1975, one of the three related patients presented with colon polyps, of which one progressed to carcinoma [14]. Since then, there has been debate about whether colorectal neoplasia is typically associated with BHDS, with recent evidence suggesting an existing relationship. A study by Nahorski et al. demonstrated that somatic frameshift mutations in a specific exon in the mRNA sequence of the *FLCN* gene were detected in 23% of BHDS patients with colorectal neoplasia [15]. A study by Sattler et al. comparing the incidence of colorectal cancer in patients with versus without BHDS demonstrated similar findings, where patients from families with BHDS had a significantly higher incidence of early-onset colorectal cancer than those without BHDS [16]. These findings suggest the existence of a possible underlying genetic relationship between BHDS and colorectal neoplasia, highlighting the importance of early screening in patients which may allow for earlier identification of BHDS before the onset of more severe symptoms such as renal cancers. Our patient had a history of colon polyps and his paternal uncle was diagnosed with colorectal cancer, a finding that supports the hypothesis of colorectal neoplasia being a manifestation of BHDS. It is thus possible this patient’s parents and paternal uncle may have had undiagnosed BHDS.

## Conclusions

BHDS typically presents with fibrofolliculomas, pulmonary cysts, pneumothorax, and a greater risk of developing renal cancer. However, as demonstrated in this case, the disease can present with various dermatological and pulmonary manifestations including hydropneumothorax, bilateral pneumothorax, and actinic keratosis. While incidence is rare, it is crucial for clinicians to be aware of the multiple ways BHDS can present to ensure the disease is not prematurely ruled out. Given the autosomal dominant nature of the syndrome, it is vital that an accurate diagnosis is made to allow for proper genetic counseling along with early and routine surveillance for renal cancer, as it is the leading cause of mortality in BHDS patients. 
